# The involvement of the piriform cortex in non-lesional temporal lobe epilepsy: an uncommon component of the epileptogenic network

**DOI:** 10.1093/braincomms/fcae179

**Published:** 2024-07-16

**Authors:** Nigel P Pedersen, Ashley Raghu, Veeresh Kumar N Shivamurthy, Joshua J Chern, Robert E Gross, Jon T Willie, Raymond J Dingledine, Ammar Kheder

**Affiliations:** Department of Neurology, and UC Davis Comprehensive Epilepsy Center, University of California, Davis School of Medicine, Center for Neuroscience, Davis, CA 95618, USA; Department of Neurology, Emory University School of Medicine, Atlanta, GA 30322, USA; Department of Neurosurgery, Emory University School of Medicine, Atlanta, GA 30322, USA; Department of Neurology, Saint Francis Hospital and Medical Center, Trinity Health of New England, Hartford, CT 06105, USA; Department of Neurosurgery, Emory University School of Medicine, Atlanta, GA 30322, USA; Epilepsy Center, Children’s Healthcare of Atlanta, Atlanta, GA 30342, USA; Department of Neurosurgery, Emory University School of Medicine, Atlanta, GA 30322, USA; Department of Neurological Surgery, Washington University School of Medicine, St. Louis, MO 63110, USA; Department of Pharmacology and Chemical Biology, Emory University School of Medicine, Atlanta, GA 30322, USA; Department of Neurology, Emory University School of Medicine, Atlanta, GA 30322, USA; Epilepsy Center, Children’s Healthcare of Atlanta, Atlanta, GA 30342, USA

**Keywords:** piriform cortex, temporal lobe epilepsy, stereo-electroencephalography, SEEG, olfactory aura

## Abstract

The piriform cortex is recognized as highly epileptogenic in rodents, yet its electrophysiological role in human epilepsy remains understudied. Recent surgical outcomes have suggested potential benefits in resecting the piriform cortex for cases of medial temporal lobe epilepsy. However, little is known about its electrophysiological activity in human epilepsy. This case-series study aimed to explore the electrophysiological role of the piriform cortex within the epileptogenic network among patients with suspected temporal lobe epilepsy. Participants were recruited from Emory University Hospital or Children’s Healthcare of Atlanta, with non-lesional frontotemporal or temporal lobe hypotheses, undergoing stereoelectroencephalographic studies. Specifically, focus was placed on patients with one or more electrode contacts in the piriform cortex. Primary objectives included determining piriform cortex involvement within the electrophysiologically defined epileptogenic network and assessing the effects of electrical stimulation. Twenty-two patients were included in the study. Notably, only one patient exhibited piriform cortex involvement at seizure onset, associated with an olfactory aura. Two patients showed early piriform cortex involvement, while others displayed late or no involvement. Electrical stimulation of the piriform cortex induced after-discharges in three patients and replicated a habitual seizure in one. These findings present a contrast to surgical outcome studies, suggesting that the piriform cortex may not typically play a significant role in the epileptogenic network among patients with non-lesional temporal lobe epilepsy.

## Teaser

Pedersen *et al*. studied the piriform cortex’s role in human epilepsy, focusing on suspected temporal lobe epilepsy cases. Twenty-two patients underwent stereoelectroencephalographic studies with electrodes in the piriform cortex. Contrary to surgical outcome studies, results indicate limited piriform cortex involvement, challenging its presumed significance in non-lesional temporal lobe epilepsy.

## Graphical Abstract

**Graphical Abstract:**
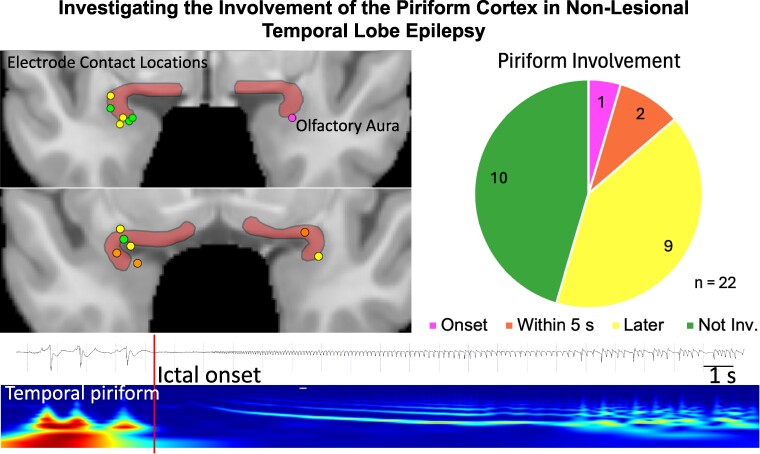

## Introduction

The piriform cortex (PC) has garnered significant attention in the context of medial temporal lobe epilepsy (mTLE), primarily driven by three key lines of evidence. Firstly, in rodent models of epilepsy, the PC exhibits remarkable hyperexcitability. It is susceptible to electrical kindling and can serve as a site for spontaneous seizure initiation in systemic chemoconvulsant models.^1^ Secondly, incorporating the PC into temporal lobe resections for patients with mTLE has shown notable improvements in seizure outcomes, especially in cases of lesional temporal lobe epilepsy.^2-4^ Lastly, the occurrence of olfactory auras accompanying seizures in mTLE implicates olfactory cortices, encompassing the cortical nucleus of the amygdala and the PC.^5^ Anecdotally, during stereoelectroencephalographic (SEEG) studies, we observed that PC stimulation infrequently leads to after-discharges. Furthermore, the PC was seldom identified as part of the epileptogenic network in patients with pre-implantation temporal lobe hypotheses.

Building upon these anecdotal insights, we formulated the hypothesis that the PC’s hyperexcitability in humans might not be as pronounced as in rodents, and it might rarely contribute to the epileptogenic network. To investigate this, our study compiled cases of patients with electrode contacts in the PC region.

## Materials and methods

Patients with medically refractory epilepsy undergoing pre-surgical evaluation with SEEG in our centre from 2016–21 were retrospectively reviewed under the approval of our Institutional Review Board. The study included patients who were suspected to have an epileptogenic network involving the medial temporal lobe. The decision to implant the piriform cortex electrode was tailored to patients exhibiting specific characteristics indicative of mesial temporal lobe involvement, based on pre-surgical data. Additionally, these patients had one or more electrode contacts in the region encompassing the temporal piriform (tPC) or the lateral olfactory area (formerly referred to as the ‘frontal piriform area,’ fPC).^6^ The confirmation of electrode placement was achieved through neuroimaging. Contacts up to 5 mm distant from the nuclei were included, safely within the accepted range of field potential recording.^7^

**Figure 1:**
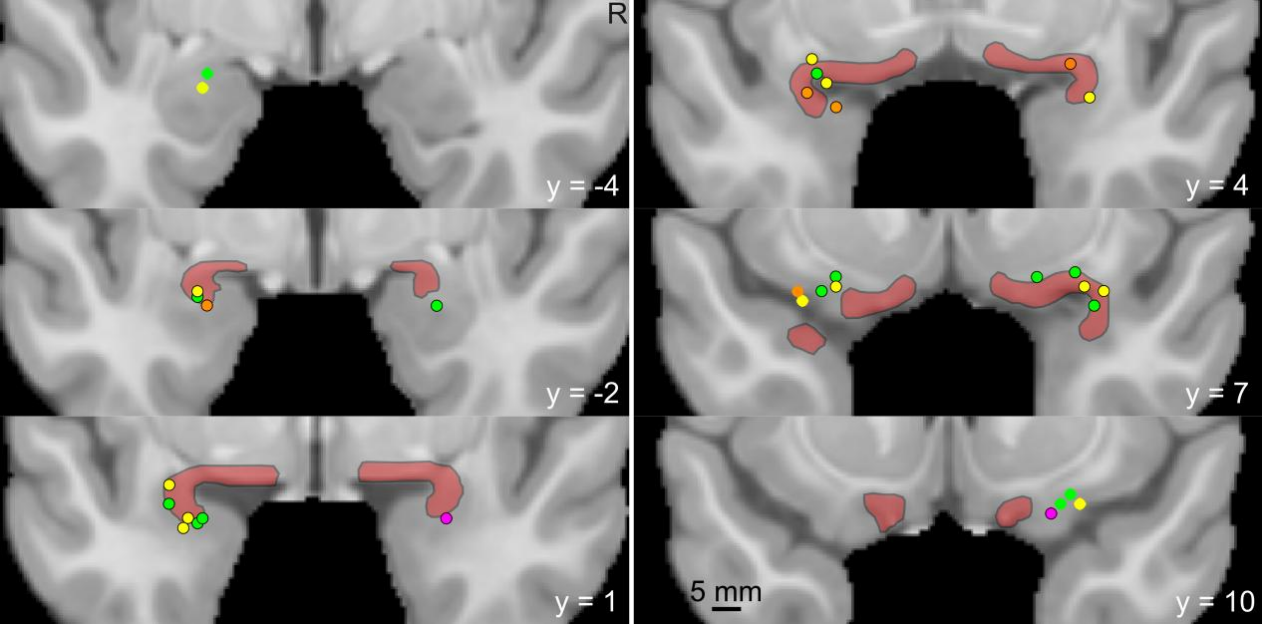
**The piriform cortex and location of electrode contacts.** The piriform cortex is delineated with back margins and transparent red infill according to published boundaries on an MNI template brain.^9^ Electrode contacts from all patients are shown as coloured circles. A black border on the circle indicates that the contact is close enough to pick up activity within the piriform region. Magenta circles denote those involved at ictal onset, orange as part of the early ictal network (<5 s), yellow as involved later in the seizure, and green contacts were not involved in patient seizures. The anterior–posterior coordinate in MNI space is shown as the *y* coordinate.

All patients had a surgical intervention or neuromodulator implantation, adequate neuroimaging for localization, and Engel outcomes at 18 months or more. Outcomes after surgery provided additional evidence for determining the underlying seizure network anatomy for this study. MRI studies were reviewed by a trained neuroradiologist and again by the authors (A.K., V.K.N.S.), to determine whether mesial temporal sclerosis (MTS) was present. For each patient, a postoperative CT image was registered to a preoperative 3T MP-RAGE image sequences, which were, in turn, non-linearly registered to Montreal Neurological Institute (MNI) template.^8^ To aid visualization, a PC mask was generated based on the method of Zhou *et al*.^9^ The attribution of SEEG contacts to the PC region was performed jointly by N.P.P., ALBR and A.K., based on ‘leads-in’ MR, fused CT-MR, MNI-normalized electrode artefacts and with reference to atlases, including both a classical and corrected modern demarcation of the PC^6,10^ (Fig. 1). A mean of four seizures was reviewed per patient. Three epileptologists reviewed raw EEG data to reach a consensus. Electrographic and semiologic correlations were studied in 5-s intervals to evaluate early versus late involvement of the temporal or frontal PC (tPC and fPC). EEG data included interictal activity in the PC, seizure onset and the pattern of onset [rhythmic spikes, onset as or rapid evolution to low voltage fast activity (LVFA), or other types]. We further categorized the involvement of the PC in seizures into three phases: at onset, early (≤5 s) and late (>5 s). Finally, we conducted anatomo-electrical clinical correlations of seizures, including an analysis of semiologic features associated with propagation to the PC.

### Statistical analysis

Descriptive statistics (means and percentages) summarized demographic and clinical characteristics. Comparative statistical analysis has been conducted to analyse patient outcomes. Analyses were performed using Excel® for Microsoft 365 MSO (Version 2312).

## Results

We identified a total of 22 patients who met the aforementioned criteria, comprising 14 females and 8 males. None of these patients had exhibited signs of MTS, and detailed patient information is provided in Table 1.

**Table 1:** Patient characteristics

Pt	Age	Sex	tPC	fPC	IEDs	Onset site	PC SO	Latency (s)	MRI findings	Procedure	PC+/−	Engel	Follow-up (months)
1	28	M	Y	Y	N	Amy, HC	N	3	Parietal leukomalacia	LITT	−	1B	54
2	37	F	Y	N	N	Amy, HC	N	NI	Negative	LITT	−	1A	45
3	32	F	Y	N	Y	HC, MTG	N	NI	Negative	ATL	+	3A	25
4	34	M	Y	N	Y	Ant. Cing	N	70	Negative	Frontal Res	+	2	18
5	25	F	Y	N	N	Basal temporal	N	6	TE	LITT	+	1A	22
6	27	F	Y	N	Y	Ant Cing	N	NI	Negative	LITT	−	2	60
7	37	F	Y	N	N	EC, HC	N	NI	TE	LITT	−	1A	42
8	56	M	Y	N	N	Basal frontal	N	NI	Bilateral hippocampal atrophy	LITT	+	1B	38
9	36	F	Y	Y	N	HC, PHG	N	NI	Negative	DBS	−	2	43
10	26	M	Y	N	Y	Anterior temporal	N	10	Negative	ATL	−	1A	34
11	27	F	Y	N	Y	Bitemporal	N	NI	Negative	RNS	−	3	18
12	33	F	N	Y	N	Bitemporal	N	11	Negative	RNS	−	3	22
13	33	M	Y	N	Y	Amy, HC	N	20	TE	LITT	−	1A	48
14	38	M	Y	Y	Y	Anterior temporal	N	4	Negative	LITT	−	1A	19
15	29	M	Y	N	Y	Amy, HC	N	NI	Negative	LITT	−	1A	35
16	40	M	N	Y	N	Basal temporal	N	NI	Negative	LITT	−	1A	20
17	19	F	Y	Y	Y	Anterior temporal	Y	Onset	Negative	ATL	+	1A	27
18	43	F	N	Y	N	Amy, HC	N	NI	Negative	LITT	−	3	32
19	39	F	Y	N	N	Anterior temporal	N	Late	Negative	LITT	−	3	33
20	41	F	N	Y	N	Anterior temporal	N	Late	Negative	LITT	−	2	38
21	33	F	Y	Y	N	OFC	N	NI	Negative	LITT	−	1A	23
22	36	F	Y	N	N	Anterior temporal	N	Late	Negative	LITT	−	1A	30

tPC, temporal piriform area; fPC, frontal piriform area (lateral olfactory nucleus); IEDs, interictal epileptiform discharges in PC; PC SO, PC seizure onset. Latency in seconds from seizure onset. Network: Broad categorization of the epileptogenic network. Procedures include responsive neurostimulation (RNS), deep brain stimulation (DBS), laser interstitial thermal therapy (LITT) and resection (RES). PC+/− refers to the PC being intact (+) or either resected or ablated (−). Engel classes I–VI are given for all patients. NI, not involved; TE, temporal encephalocele.

**Figure 2:**
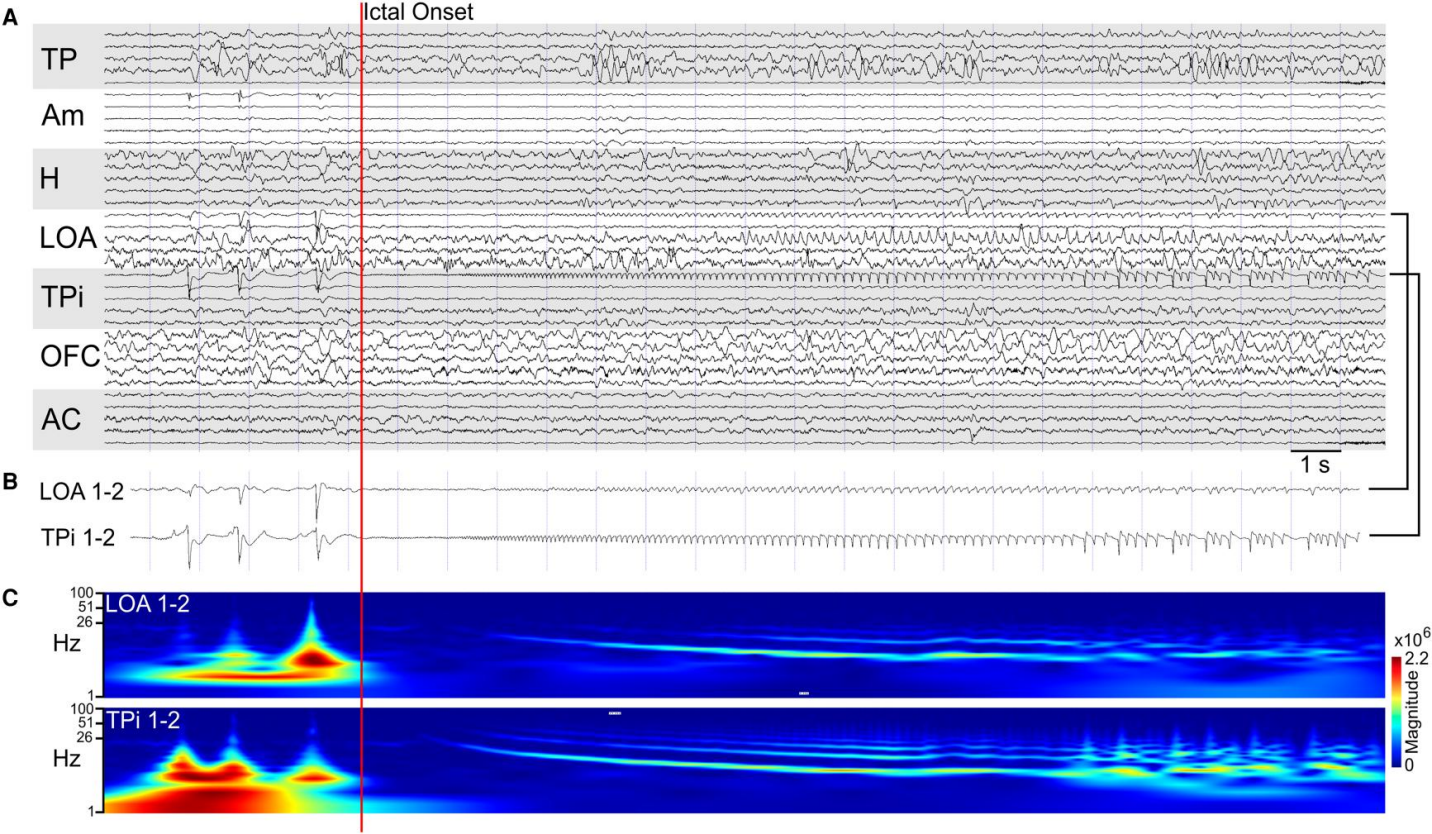
**Electrophysiology of a seizure starting in the piriform cortex.** (**A**) An intracranial recording in a bipolar montage from patient 17. Electrodes are labelled based on the anatomical location of medial contacts at the electrode tip (TP, temporal pole; Am, amygdala; H, hippocampus; LOA, lateral olfactory nucleus/frontal piriform; TPi, temporal piriform; OFC, orbitofrontal cortex; AC, anterior cingulate). (**B**) Magnified view of the channels that show low voltage fast activity at ictal onset. (**C**) Wavelet (Morlet) spectrogram of the contacts shown in **B** over the same time period.

Intermittent interictal spikes, occurring at a frequency of a few per hour, were observed in the PC of eleven patients. Specifically, spikes in the fPC were noted in two patients, although this region did not play a role in the early ictal onset. In contrast, spikes in the temporal piriform cortex (tPC) were present in nine patients, with two displaying early ictal involvement. Notably, one patient exhibited a seizure onset (SO) characterized by LVFA that encompassed the tPC (Fig. 2). Among those without PC involvement at the onset of seizures, two patients demonstrated PC involvement within 5 s, and two others within 10 s. However, there was no ictal involvement noted in the remaining patients. Importantly, the three patients with onset or early involvement of the PC constituted a subset of 16 patients whose seizures onsets included other medial temporal lobe structures. Based on SEEG findings and electroclinical correlations, 18 patients were diagnosed with temporal lobe epilepsy, of which two had bitemporal involvement. Additionally, four patients were diagnosed with extratemporal epilepsy, involving either the frontal or frontotemporal network. Notably, early PC involvement was consistently associated with concurrent spread to the anterior insular cortex across all three patients. Semiological analysis revealed an olfactory aura in the one patient with tPC onset; however, no other specific semiologic correlates were identified at the time of PC involvement in the remaining patients. Furthermore, high-frequency cortical stimulation of the PC (50 Hz, 300 ms, 5 s, 3 mA) resulted in a habitual seizure in one patient and local after-discharges in three patients. All patients underwent therapeutic procedures, as detailed in Table 1. Specifically, three patients underwent neuromodulatory therapy, involving responsive neurostimulation (RNS) for two patients and open-loop deep brain stimulation (DBS) for one patient. Additionally, 15 patients with temporal lobe epilepsy underwent ablation or resection procedures, with 12 out of 15 (80%) achieving favourable Engel class I outcomes (*n* = 11) or class II outcomes (*n* = 1). Importantly, the Engel outcomes of patients who underwent PC ablation or resection did not differ significantly from those with an intact PC in this series.

Supplementary Figs 1 and 2 depict examples of ablation and resection, highlighting interventions that spare (Fig. 1) and disconnect (Fig. 2) the PC region.

## Discussion

This study represents the first case series examining the electrophysiology of the piriform cortex in human epilepsy. Our findings suggest that the involvement of the PC in the early ictal network is relatively infrequent, even among patients diagnosed with mTLE. Notably, one patient who exhibited seizure onset within the piriform cortex reported an olfactory aura, which aligns with the established role of the PC in olfaction.^11^

Our study also reveals that the insular cortex becomes engaged in seizure propagation when the PC is involved near or at the onset of seizures. This observation is consistent with the concept that the insula, in conjunction with the amygdala, plays a central role in human olfactory networks.^12^ On the surface, these findings may appear contradictory to the demonstrated epileptogenic role and hyperexcitability of the PC in rodent epilepsy models.^1^ Additionally, our results differ from the implications drawn from recent epilepsy surgery outcome studies, which examined the potential benefits of incorporating the PC into temporal resections or ablations.^2-4^

Recent clinical investigations have indicated that resecting or ablating the PC is associated with improved outcomes in patients with temporal lobe epilepsy. Influenced by the high epileptogenicity of the PC in rodent models, a multicentre study, primarily involving patients with identifiable lesions, who had undergone anterior temporal lobectomy, found a correlation between the extent of PC resection and achieving seizure freedom.^2^ The odds ratio for achieving seizure freedom with more than 50% PC resection was 16 (95% CI 5–47). Of note, the extent of resection of the amygdala, hippocampus and entorhinal cortex varied significantly in this study but did not differ between patients who became seizure-free and those who did not. Total resection volume was also comparable between the two groups. These results led to the inference that the PC plays a pivotal role in ictal onset. Subsequent studies replicated these findings, focusing on patients selected for transylvanian selective amygdalo-hippocampectomy, and again highlighted that the extent of PC resection, rather than other medial temporal lobe structures, correlated with a higher rate of seizure freedom.^3,4^ It’s important to note that none of these studies involved intracranial recordings of the PC, and most of the patients had medial temporal sclerosis (MTS).

The results of our study complement existing knowledge and contribute to our understanding of the human PC. The most noticeable distinction between our study and previous outcome-based research is the absence of MTS in our patient cohort. Studies have indicated that smaller MRI volumes of the piriform cortex are linked to the duration of epilepsy and the presence of MTS in mTLE.^13^ Moreover, in cases of human poisoning with domoic acid, significant cell loss and injury to the PC are observed, closely mirroring findings in rodent chemical kindling models.^14^ It’s important to note that mTLE is not a uniform condition, and preclinical and clinical evidence suggests the existence of diverse epileptogenic networks in temporal lobe epilepsy.^15^

In light of these previously reported insights, coupled with our recent observations, we propose that the role of the piriform cortex in epilepsy may be critical in mTLE cases accompanied by MTS, but this might not be the case in non-lesional mTLE and TLE.

Our study exhibits certain limitations. Firstly, it is a retrospective study conducted across two institutions, characterized by a relatively small sample size. This may constrain the applicability of our findings to a more extensive range of patients with temporal lobe epilepsy. Additionally, the absence of a control group, comprising patients with medial temporal sclerosis but without piriform cortex involvement, introduces challenges in making definitive conclusions regarding the distinct contribution of the PC.

## Conclusions

Our findings demonstrate that the piriform cortex is infrequently integrated part of the epileptogenic network in a series of patients with non-lesional temporal lobe epilepsy. As anticipated, the early involvement of the PC can be associated with an olfactory aura. We propose that the PC might assume a more substantial role in cases of mTLE accompanied by MTS. Further investigations are warranted to gain a deeper understanding of the precise role played by the PC in mTLE cases co-occurring with MTS.

## Supplementary Material

fcae179_Supplementary_Data

## Data Availability

The data that support the findings of this study are available from the corresponding author, A.K., upon reasonable request.
