# Supplementary material for: The involvement of the piriform cortex in non-lesional temporal lobe epilepsy: an uncommon component of the epileptogenic network
Source: Brain Commun. 2024 Jul 16;6(4):fcae179. doi: 10.1093/braincomms/fcae179 (PMC11249973; doi:10.1093/braincomms/fcae179)
Supplement: fcae179_Supplementary_Data [file fcae179_supplementary_data.pdf]

Supplementary Figures

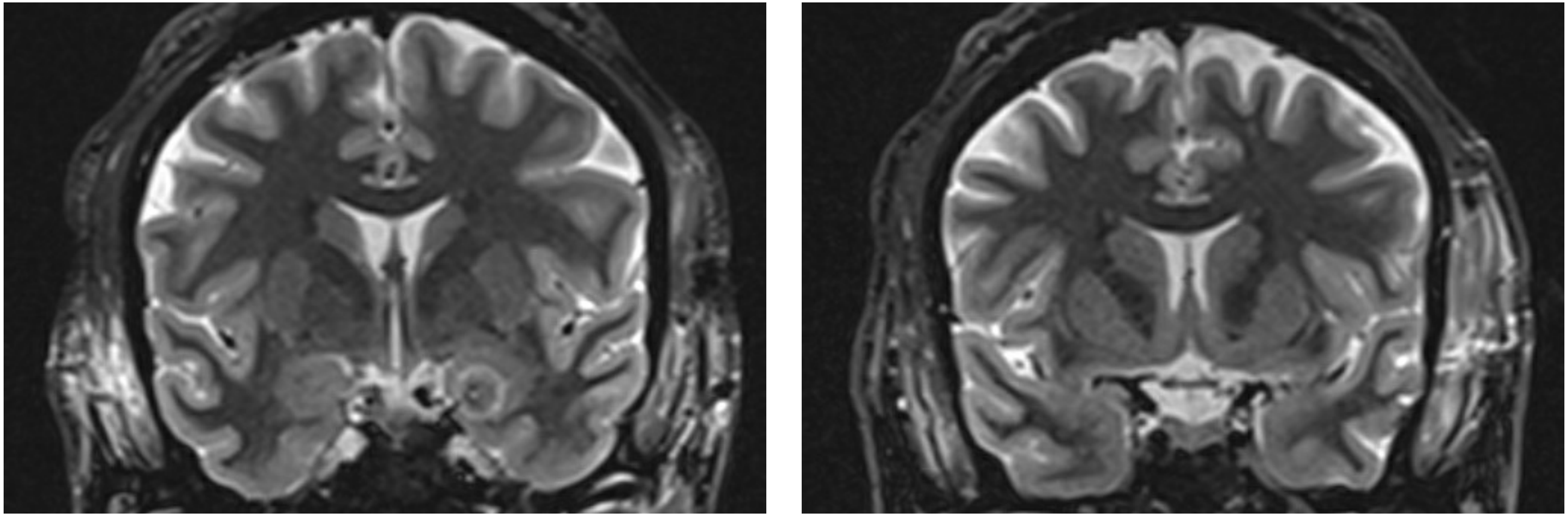

Supplementary Figure 1: Coronal T2 MRI of Stereotactic laser amygdalohippocampotomy (SLAH) of the left medial temporal structure sparing the Piriform Cortex in patient 2

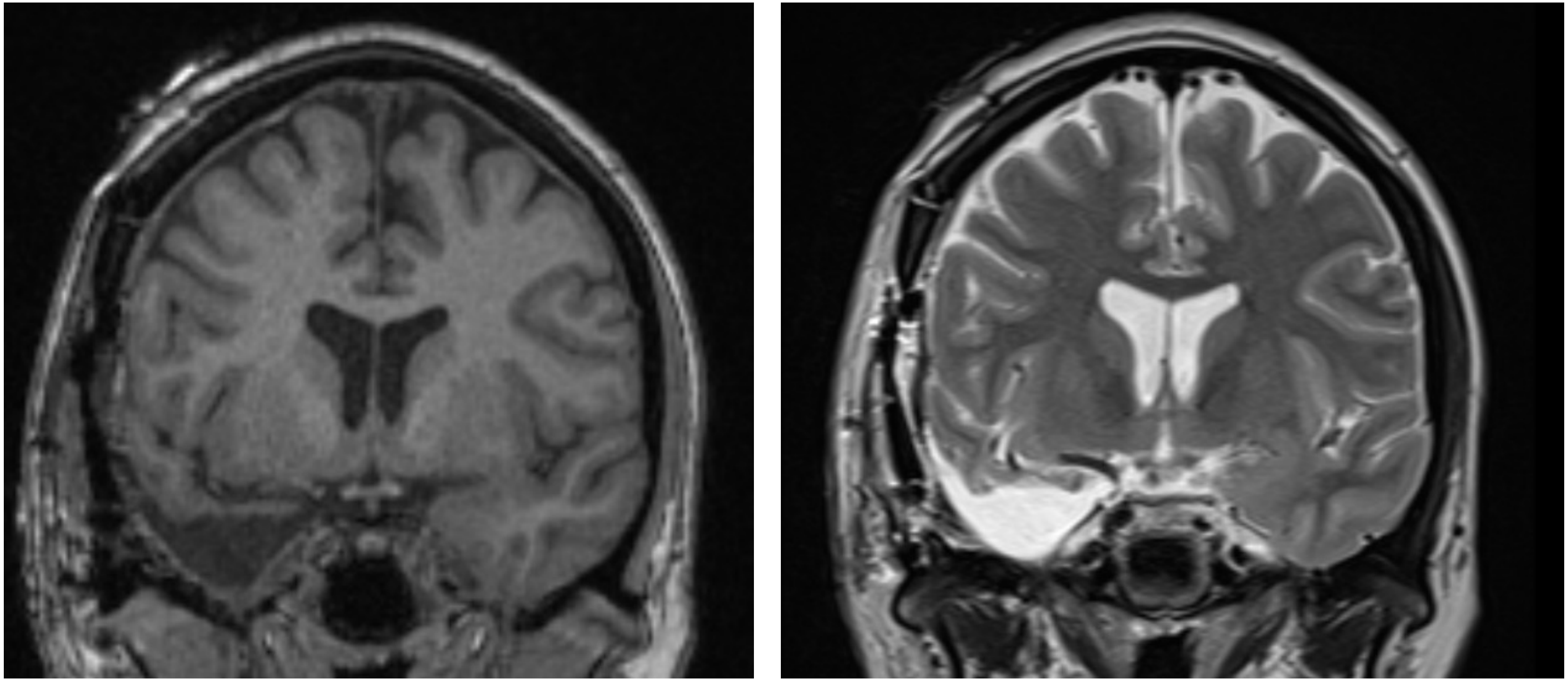

Supplementary Figure 2: Coronal postoperative MRI in subject 4 showing disconnection of the Piriform Cortex
